# Improved Laccase Production by *Trametes pubescens* MB89 in Distillery Wastewaters

**DOI:** 10.4061/2011/379176

**Published:** 2011-11-30

**Authors:** P. J. Strong

**Affiliations:** ^1^Product Recovery, LanzaTech, 24 Balfour Road, Auckland 1052, New Zealand; ^2^Department of Biochemistry, Microbiology and Biotechnology, Rhodes University, P. O. Box 94, Grahamstown 6140, South Africa

## Abstract

Various culture parameters were optimised for laccase synthesis by *Trametes pubescens* MB89, including pH, carbon source, nitrogen source, lignocellulosic supplements, and reported inducers. Glucose, in conjunction with a complex nitrogen source at pH 5.0, resulted in the highest laccase yield. Adding ethanol, copper, or 2,5-xylidine prior to inoculation further improved laccase concentrations. The addition of 2,5-xylidine was further investigated with multiple additions applied at varying times. This novel application substantially improved laccase production when applied regularly from inoculation and during the growth phase, and also countered glucose repression of laccase synthesis. Single and multiple factor changes were studied in three distillery wastewaters and a wine lees. A synergistic increase in laccase synthesis was observed with the addition of glucose, copper, and 2,5-xylidine. Single addition of 2,5-xylidine proved most beneficial with distillery wastewaters, while copper addition was most beneficial when using the wine lees as a culture medium.

## 1. Introduction

Laccase and various microorganisms that produce the enzyme have been studied intensively due to their potential applications in industrial and remediative processes. However, one of the factors inhibiting the application of laccase is the cost associated with using large quantities of the enzyme. A possible strategy is to improve laccase yields using waste substrates as a culture media for solid or submerged fermentations. Numerous studies have investigated the most favourable conditions for laccase production by various fungi with solid and submerged fermentations [1]. The production of laccase is dependent on a number of factors, which include the strain of microorganism (or genetic manipulation thereof), the composition of the culture medium (compounds that provide a nitrogen and carbon source or that act as inducers), the cultivation method (solid substrate or submerged), and the culture conditions (oxygen availability, pH, temperature). Laccase is generally produced in appreciable concentrations during the idiophase, where growth remains static due to a decrease in available substrate, but may be significantly enhanced by adding inducer compounds. In order to provide laccase in the quantities required and at a low cost, it is vital that yields are increased or that production costs are reduced [2]. 

A variety of agroindustrial waste residues may be utilized to produce laccase and thereby lower the substrate costs involved in production. Barley bran [3], a common waste from the brewing industry, chestnut shell waste from glacé chestnut production [4], banana skins [5], mandarin peels [6], kiwi fruit wastes [7], grape seeds [8], and distillery wastewaters [9] have all been assessed as substrates for laccase synthesis using white rot fungi. Distillery wastewaters are particularly attractive for monocultures as they can be considered as a sterilised growth medium, notably lowering costs associated with heat sterilisation. Although these waste substrates have been investigated as potential substrates for laccase production, this needs to be taken further and laccase yields using waste residues need to be increased. Minor adjustments to the culture conditions, supplementation, or inducer addition could significantly improve laccase production when utilizing waste substrates. 

Inducers are compounds that significantly increase laccase production while occurring at concentrations that are extremely low relative to available carbon sources. Many inducers are phenolic or aromatic compounds related to lignin or are lignin derivatives. Non-phenolic compounds such as ethanol [11] and metal ions such as copper [12, 13] have also increased laccase synthesis. The presence of the inducer (or possibly its metabolite) and the availability of copper can trigger significant increases in laccase productivity in response to environmental conditions. The extent to which laccase synthesis is enhanced depends upon the inducer's concentration and its time of addition [1]. If it occurs at too low a concentration then no effect is observed, while a toxic effect (growth inhibition) is often observed when the concentration is too high. Although the time at which as inducer is added does affect laccase synthesis, the majority of studies add the compound prior to inoculation. Fungal genera differ markedly regarding laccase stimulus by inducers. The inductive effect also depends on very small differences in molecular structure, as large differences in enzyme synthesis have been noted for very similar compounds [10]. 

The objective of this study was to enhance laccase synthesis by *Trametes pubescens *MB89 with pH adjustment, carbon and nitrogen supplementation, and the addition of a variety of reported inducers at two time periods. The most stimulatory compound was then assessed further using different numbers of additions, at different times to determine which would have the greatest positive impact on enzyme yields. The changes or additions that resulted in increased laccase synthesis were assessed in wine-related wastewaters to establish whether the improvements would have a universal effect or if they were particular to a specific culture medium.

## 2. Materials and Methods

### 2.1. The Effect of pH, Different Carbon, Nitrogen, and Lignin/Cellulose Substrates on Laccase Synthesis

The optimal pH was assessed using a full-strength distillery wastewater (COD 29.5 g/L, total phenolic compounds 280 mg/L, and pH 3.75) adjusted to 3.5, 4.0, 4.5, 5.0, 5.5, and 6.0 using hydrochloric acid or Na_2_CO_3_ powder (both Saarchem, uniLAB, Merck). Aliquots of 65 mL of the wastewater were placed in 250 mL Erlenmeyer flasks, covered with aluminium foil (to prevent contamination), and autoclaved for fifteen minutes. Duplicate flasks were inoculated with *T. pubescens* MB89 (0.87 ± 0.28 g/L) from stock cultures grown in a liquid culture containing 2% malt extract, 1% glucose, and 0.2% yeast extract (all Merck, Biolab) at pH 5.5. 

Different carbon sources in the form of fructose, glucose, mannitol, maltose, sucrose, cellobiose, and lactose (all Saarchem, univAR, Merck) were added to a low-strength brandy distillery wastewater (COD 10.5 g/L, total phenolic compounds 35 mg/L, and pH 3.9) to assess their individual effects on laccase synthesis. The amount added was equivalent to the molar equivalent of carbon atoms in 10 g/L of glucose. Different nitrogen sources in the form of NH_4_NO_3_, NH_4_Cl, KNO_3_ (Saarchem, univAR, Merck), and H_2_NCNH_2_ (analaR, BDH) were added at a molar equivalent of nitrogen atoms in 2 g/L of KNO_3_, while malt extract, yeast extract, and peptone were added at 2 g/L. Cellulose and lignin-containing supplements in the form of cellulose powder, blue gum powder, rooibos tea leaves (*Aspalathus linearis*), and sugarcane bagasse were added at 1 g/L, and phosphorus (H_3_PO_4_, 50 mM) was assessed. In all cases, the wastewater pH was adjusted to 5.0 using sodium carbonate powder. Aliquots of 65 mL of the solutions were placed in 250 mL Erlenmeyer flasks, covered with aluminium foil (to prevent contamination), and autoclaved for fifteen minutes. Triplicate flasks were inoculated with *T*.* pubescens* MB89 (1.27 ± 0.31 g/L) from the stock cultures described above. The flasks were placed in a shaking incubator (Labcon) at 150 rpm at 28°C for 15 days. Control samples were inoculated in the media containing no stimulatory compounds. Samples were taken from the flasks at least every second day, centrifuged in 1.5 mL Eppendorf containers at 9660 g for two minutes (Heraeus Biofuge, Germany) and the supernatant was diluted appropriately and tested for laccase activity using the ABTS assay as described in [9].

### 2.2. The Effect of Reported Inducers

#### 2.2.1. Addition Prior to Inoculation

All inducers were assessed in 250 mL Erlenmeyer flasks containing 65 mL of a synthetic medium containing: 2% glucose (Saarchem, uniLAB, Merck), 0.3% peptone, 0.3% malt extract (both Biolab, Merck), KH_2_PO_4_ (1 g/L), Na_2_HPO_4_·2H_2_O (100 mg/L), MgSO_4_·7H_2_O (500 mg/L), CaCl_2_ (10 mg/L), FeSO_4_·7H_2_O (10 mg/L), MnSO_4_·4H_2_O (1 mg/L), ZnSO_4_·7H_2_O (1 mg/L), and CuSO_4_·5H_2_O (2 mg/L) (all Saarchem, uniLAB, Merck). Reported inducers in the form of 3,4-dimethoxybenzyl alcohol, 2,5-xylidine (2,5-dimethylalinine), syringic acid, hydroxybenzotriazole (HBT), violuric acid (all Fluka, Sigma Aldrich Ltd, Cape Town), guaiacol, *p*-coumaric acid, 2,6-dichloroindophenol (DI), quercetin dehydrate,* o*-cresol, gallic acid (all Sigma), *n-hydroxyphthalimide*, 4-methylcatechol (both Aldrich), phenol, phenol red, and copper sulphate (all Saarchem, uniLAB, Merck) were all tested at 1 mM, while cycloheximide (Sigma-Aldrich), an antibiotic, was tested at 0.1 mM. Tannic acid (Sigma), cellulose powder (Aldrich, 20 micron diameter), *Aspalathus linearis* tea leaves, and absolute ethanol (Merck) were tested at 0.1% (w/v). All reported inducers, other than ethanol, were autoclaved in the synthetic medium (pH adjusted to 5.0 individually after the addition of the inducer). Absolute ethanol was autoclaved separately and added immediately prior to adding the inoculum.

#### 2.2.2. Addition Four Days after Inoculation

Autoclaved flasks containing only the synthetic medium described in Section 2.2.1 were inoculated and placed on a shaking incubator (Labcon) at 150 rpm at 28°C. After four days of growth, the reported inducers were added individually under aseptic conditions. All reported inducers and controls were assessed in triplicate over a 20-day period. Samples of <0.5 mL were taken every 48 hours, except for 2,5-xylidine—which was sampled daily for the first six days after addition and every 48 hours thereafter.

#### 2.2.3. Multiple Additions of 2,5-Xylidine

One reported inducer, 2,5-xylidine, was additionally tested in the synthetic medium by varying the both time and number of additions. It was added aseptically such that the concentration increased by 1 mM with each addition. One, two, or three doses were administered at 48-hour intervals. Dosing commenced at different times after inoculation (see Table 3, Section 3.2.2 for the exact times and numbers of addition) to determine the effects of dosing during different stages of the growth cycle.

### 2.3. Laccase Synthesis in Modified Wine-Related Wastewaters

Four wastewaters were obtained from a winery and two distilleries near Worcester in the Western Cape Province of South Africa and stored at 4°C. After assessment for growth inhibition, two distillery wastewaters were tested at full strength, while two of the wastewaters (a wine lees and a distilled wine lees after tartaric acid extraction) were tested at 30% concentration. Wastewater controls consisted of the raw, unadjusted wastewater. Additions of 2% glucose, 1 mM copper sulfate, or three 1 mM additions of 2,5-xylidine were assessed in the four wastewaters. In addition, a synergistic reaction was studied by combining pH adjustment, glucose, copper, and 2,5-xylidine addition. Triplicate flasks of all wastewaters were inoculated with biomass of *Trametes pubescens *MB89 (0.76 ± 0.25 g/L) from the stock cultures described above. The flasks containing the wastewater samples were placed on a benchtop shaker (Labcon SP015 + UPF75, Maraisburg) at 150 rpm at 28°C for 14 days. Laccase activities were tested every 48 hours for all flasks except for 2,5-xylidine, which was tested every 24 hours for the first eight days and every 48 hours thereafter.

## 3. Results and Discussion

### 3.1. The Effect of Different Carbon, Nitrogen, and Lignin/Cellulose Substrates on Laccase Synthesis

#### 3.1.1. pH

**Figure 1:**
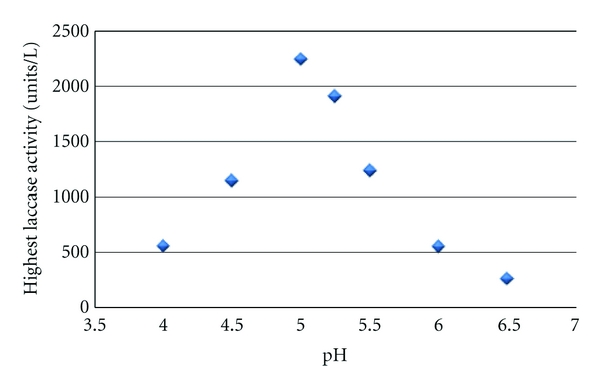
The highest laccase concentration produced at various starting pH values (*n* = 2).

Laccase synthesis varied significantly over the pH range tested in the brandy distillery wastewater. A peak in production was evident at pH 5.0—as laccase synthesis decreased by more than 40% at a pH only 0.5 units more acidic and basic (Figure 1). Variations in growth and metabolic requirements could be attributed to the change in laccase production. A visibly longer lag phase in growth occurred at more acidic pH values, and less mycelia growth was evident. The optimal range for the laccase isoforms secreted by this fungal strain has been reported between pH 3.0 and 4.5 [12], potentially indicating that laccase may be produced and function optimally under conditions that are not favourable to growth. The presence of inducers may increase laccase synthesis by providing contact with compounds that may naturally elicit a stress response and further increase production. In the current experiment, all of the highest laccase activities occurred from day 12 onwards, which is typical of many submerged cultures where the highest activities were reported to occur in the secondary growth phase.

#### 3.1.2. Carbon Source

The greatest laccase synthesis was obtained when fructose, glucose, sucrose, and cellobiose were used as carbon sources (Table 1). These sugars all improved laccase yield 1.7-fold relative to the control. Lactose and maltose also yielded relatively high laccase production and improved synthesis 1.5-fold. Mannitol was the only supplement that resulted in a relative decrease in laccase synthesis, as these cultures produced approximately half the laccase produced in the control. Mannitol differed from the other carbon sources in that it was a sugar alcohol (or polyol) and was not a cyclic compound. Revankar and Lele [14] investigated the effect of different carbon sources (glucose, fructose, sucrose, lactose, starch, and glycerol) on laccase production by *T. versicolor *MTCC138. They observed a 3-fold increase of laccase production when glucose was used instead of fructose, and starch further improved laccase production by 12%. Although not attempted in this study, Revankar and Lele [14] obtained interesting results when combining starch and glucose (1 : 1) as carbon sources and further improved laccase synthesis by 57%. They attributed lower laccase synthesis with glucose alone due to glucose repression of enzyme synthesis but when used in combination with a more complex carbon source, the glucose was rapidly utilized for growth, while the starch was consumed during stationary phase and aided laccase production.

#### 3.1.3. Nitrogen Source

The nitrogen source that improved laccase synthesis to the greatest extent was peptone (1.8-fold increase). Lower yields were obtained with an inorganic nitrogen source. The effects of inorganic nitrogen upon laccase synthesis in this study were corroborated by Revankar and Lele [14], who obtained highest laccase activities by *Trametes versicolor* MTCC 138 using a complex nitrogen source (yeast extract) and also obtained low laccase activities when using inorganic nitrogen sources. In the present study, improved growth resulting from the carbon present in peptone may have improved total laccase synthesis. 

Although an inorganic nitrogen source such as asparigine aids downstream processes such as enzyme extraction and purification [15], researchers have observed negligible growth and laccase secretion when they replaced the complex nitrogen source with asparigine as the sole nitrogen source for *Trametes pubescens *[13]. Literature exists supporting both high [13] and low [16] nitrogen concentrations for enhancing laccase synthesis, but a high nitrogen concentration is generally favoured [1]. This has also been demonstrated at a molecular level, where increasing the nitrogen concentration in cultures of *Trametes versicolor* increased in laccase gene transcription levels [17].

#### 3.1.4. Lignin/Cellulose

*Trametes pubescens* MB89 did not increase laccase synthesis in response to the four lignin/cellulose additions. The main constituent of green rooibos (*Aspalathus linearis*, used to make a herbal tea) is dihydrochalcone aspalathin, but it is also known to contain hydroxylated benzoic and cinnamic acids, the flavonoids: luteolin, chrysoeriol, quercetin, and isoquercetin [18, 19]. Sugarcane bagasse is known to contain an array of phenolic compounds, which includes both ferulic acid and *p*-coumaric acid [20]—both known to be potent laccase inducers in some white-rot fungi [21]. Although plant extracts and lignocellulosic wastes do contain tannins and phenolic compounds known to enhance laccase synthesis [21, 22], the concentrations in the liquid media may have been too low to elicit a response.

### 3.2. The Effect of Reported Inducers

#### 3.2.1. Addition Prior to or Four Days after Inoculation

A synthetic media was used to assess a number of reported inducers (Table 2) that were either added before inoculation or four days thereafter. The greatest increase in laccase synthesis for both times of addition resulted from 2, 5-xylidine. Generally, the greatest increases in laccase synthesis were observed when the stimulatory compounds were added to the medium prior to inoculation. Ethanol and copper were most beneficial when added prior to inoculation, while 4-methylcatechol and *n-hydroxyphthalimide* resulted in greatest increases when added four days after inoculation. Gallic acid, tannic acid, and quercetin led to a modest improvement in laccase activities when added prior to inoculation. The other compounds elicited no significant improvement, or a negative response regarding laccase production. Addition of the reported inducer prior to inoculation could be more effective than addition after the biomass is actively growing, as it effectively exposed a lower biomass concentration to the stimulatory compounds for a longer time period. This was evident for 4-methylcatechol where cell growth was visibly reduced and grew in a few large clumps instead of a slurry of fine pellets that was evident in nearly all other shake-flask cultures. The exposure to cycloheximide was fatal, as no change in the medium pH or laccase activity was observed.

There have been many studies regarding the effects of inducers using a plethora of fungal genera, species, and even strains. Differences in laccase stimulation were already observed in very early studies more than half a century ago. Fåhraeus et al. [23] studied the response of a number of fungi to various laccase inducers. These inducers improved laccase activities from various *T. versicolor *strains, but only guaiacol induced enzyme synthesis in *Stereum hirsutum*. Ethanol has improved laccase synthesis significantly when used as a carbon source [24] for a monokaryotic strain *Pycnoporus cinnabarinus* ss3. Later work by this group indicated that ethanol improved gene expression and inhibited protease activity, thereby playing an important regulatory role in laccase production by the fungus [25]. Further work performed in the current study found the combination of copper, 2,5-xylidine, and glucose enhanced laccase synthesis significantly (Section 3.3).

#### 3.2.2. Multiple Additions of 2,5-Xylidine

Following on from the success of 2,5-xylidine at greatly improving laccase titres, the effects of time of addition and multiple dosing were assessed to determine whether laccase stimulation could be improved further. The time of the initial dose and the number of additional doses were varied—as per Table 3. The highest relative increases in laccase synthesis were observed for cultures where the inducer was applied very early in the batch culture. A single dose of 1mM 2,5-xylidine at the time of inoculation improved laccase synthesis 7.7-fold. Three doses of 2,5-xylidine applied from the time of inoculation at 48-hour intervals improved laccase synthesis 10.3-fold. It was evident that multiple applications of 2,5-xylidine increased laccase synthesis to a greater extent than single applications. These increases were most beneficial when added early in the growth phase.

Numerous studies have shown 2,5-xylidine to be a potent inducer amongst a variety of fungal genera [3, 16, 26]. An 8.2-fold increase was observed in laccase synthesis by *T. versicolor *[27], while a 9-fold increase was observed when added to a culture of *Pycnoporus cinnabarinus *[28]. However, to the authors knowledge, the current study is the first that illustrates the effectiveness of pulsed dosing of an inducer, which resulted in a significant improvement in laccase activity (33%) compared to the single dose strategy.

Another significant finding was that the presence of 2,5-xylidine countered glucose repression of laccase synthesis. In the control samples, the highest laccase concentrations were observed in the stationary phase at the end of the fermentation period; which has been published in prior work [9, 29]. However, with multiple doses of 2,5-xylidine, laccase expression peaked early in the fermentation period. Some fungi, such as *Lentinus edodes*, produce high concentrations of laccase during primary growth (known as constitutive production). This is advantageous as it shortens the fermentation period [22, 26]. This is highly advantageous as it may greatly lower the costs of laccase production in submerged cultures as costs associated with energy and aeration can be significantly lowered. Additionally, potential contamination that could ruin a lengthier fermentation is less of a concern when the product is produced in a shorter time.

### 3.3. Laccase Synthesis in Modified Wine-Related Wastewaters

**Figure 2:**
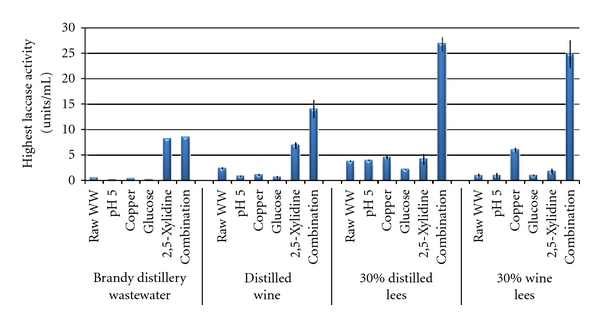
Laccase synthesis of *Trametes pubescens* in four wine-related wastewaters with various modifications (*n* = 3).

Raw wastewaters were adjusted to pH 5, supplemented with either copper, glucose, ethanol, dosed three times with 1 mM 2,5-xylidine or supplemented with a combination of glucose, copper and dosed three times with 2,5-xylidine. The initial pH, chemical oxygen demand (COD), and total phenolic compounds were measured and displayed in Table 4. The high phenolic content of the wine lees would suggest that there were likely naturally occurring phenolic inducers present. Results obtained are displayed in Figure 2, which represents the average highest laccase activity of the triplicate samples for each individual modification tested in each of the four wastewaters separately. Of the various modifications, only the addition of copper, 2,5-xylidine, or the combination of glucose, copper, and 2,5-xylidine elicited major improvements of the laccase yield.

The simultaneous addition of glucose, copper, and 2,5-xylidine resulted in the greatest increase in laccase synthesis in all four wastewaters. This would be expected as the wastewaters now contained carbon, copper (an essential metal component of the enzyme), and a reported inducer. Synergistic effects using 2,5-xylidine and other compounds have been observed before. Fåhraeus et al. [23] tested various copper concentrations with and without 2,5-xylidine and observed very little difference when copper was tested from 0 to 2500 *μ*g/L by itself. However, when copper was tested with 2,5-xylidine, there were large increases in laccase synthesis—illustrating the synergistic effect when the two inducers were added simultaneously. 

The addition of 2,5-xylidine led to the most significant increases in laccase activity in the two distillery wastewaters that were tested at full strength (Figure 2). There was no increase in the two wastewaters that were diluted to a 30% concentration. Interestingly, the flasks containing 30% wine lees that were supplemented with 2,5-xylidine were the only ones in which the fungus grew in a single mass instead of loose pellets and mycelial fragments, indicating a possible physiological response to the phenolic compound. However, laccase synthesis was not improved. The wine lees had the greatest concentration of phenolic compounds (590 mg/L after dilution), which could have acted as stimulatory compounds, and thereby lessened the effect of 2,5-xylidine. 

In the current study, laccase repression normally occurring in the presence of excess glucose was countered by the addition of 2,5-xylidine. The phenolic compound altered laccase synthesis such that the highest concentration was recorded earlier in the batch culture, as opposed to idiophasic production. When glucose was added as the only modification, the maximum laccase concentration occurred much later (approximately 11.3 days) than it did with the combination of glucose, copper, and 2,5-xylidine (approximately 6.1 days). 

Copper supplementation only increased laccase synthesis in the diluted wine lees, where the combination of the metal with organic acids, sugars, ethanol, and the various phenolic compounds could have elicited greater enzyme synthesis. Copper addition by itself was of little benefit to any of the distillery wastewaters. Copper is vital to laccase synthesis as the enzyme requires four copper atoms to be catalytically active, but lack of an adequate carbon or nitrogen source or inducer may have hampered laccase synthesis. Other studies have confirmed little difference regarding laccase synthesis by *T. versicolor *when copper by itself was tested in a range from 0 to 2500 *μ*g/L [23].

Glucose addition did not improve laccase yields. Some studies have found an abundance of glucose inhibits laccase synthesis. Moreira et al. [30] found high glucose concentrations lowered laccase production during secondary metabolism. They hypothesized that low laccase production was due to the low pH resulting from extended primary metabolism. In the current study, the biomass from the glucose-supplemented wastewaters had of a thick, clear band of mucilaginous growth (consistent with an external polysaccharide layer). When copper and 2,5-xylidine were included with glucose, this mucilaginous growth was notably diminished and the solution was less viscous. This suggested that the change in physiology during growth resulting from glucose would significantly affect laccase production; and the addition of 2,5 xylidine was beneficial with regard to both yield and downstream processing.

There was great variability in fungal growth and enzyme synthesis in wine-related wastewaters. The wine lees and the distilled wine lees had both inhibited the growth of *T. pubescens *at full strength. These had to be substantially diluted to allow for growth, which would render their use impractical at large-scale. Although there is literature pertaining to the utilisation of wastewaters as a medium or as a supplement to produce laccase [4, 21], there is little that demonstrates supplementation of wastes or wastewaters in order to enhance the production of laccase by white-rot fungi. A recent publication has illustrated that the addition of 2 mM of copper could enhance the laccase yield 3-fold using wheat bran under solid substrate fermentation [31]. The present study has shown that significant improvements can be achieved in wastewaters with slight modifications. The two distillery wastewaters that were tested at full strength had substantial increases in laccase activity when 2,5-xylidine was added by itself, while the 30% wine lees had a significant increase with the addition of 1 mM copper. The most pragmatic strategy would be to adopt a repeated dosage of 2,5-xylidine with the inclusion of a copper supplement. This would have lower associated costs, and the potential for contamination would be significantly less than if glucose were also added. Nominal copper concentrations would be added to ensure that the microelement was present as laccase synthesis is negligible if the microelement is not present. It appeared that no single factor could be altered to enhance enzyme synthesis over a broad spectrum of wine-related wastewaters. However, it was evident that supplementation with 2,5-xylidine could significantly increase laccase synthesis in full-strength distillery wastewaters.

## 4. Conclusions

Conditions tested in this study indicated that a number of factors could significantly increase laccase synthesis using *T. pubescens *in wastewaters. A pH of 5.0, a number of carbon sources (fructose, glucose, sucrose and cellobiose) and peptone all improved laccase concentrations. Under the conditions tested, 2,5-xylidine, ethanol, and copper effectively increased laccase when added prior to inoculation, while 4-methylcatechol and *n-hydroxyphthalimide* promoted laccase synthesis when added later in the growth phase. The addition of 2,5-xylidine was further investigated, and greater laccase synthesis was achieved using multiple doses applied at the early growth stage. Another significant finding was that the presence of 2,5-xylidine countered glucose repression of laccase synthesis. This is highly advantageous as it may greatly lower the costs of laccase production in submerged cultures as costs associated with energy and aeration can be significantly lowered. Additionally, potential contamination that could ruin a lengthier fermentation is less of a concern when the product is produced in a shorter time. The highest laccase concentrations were produced in all four wastewaters when the combination of copper, 2,5-xylidine, and glucose was added. The two distillery wastewaters that were tested at full strength had a substantial increase in laccase activity when supplemented with 2,5-xylidine, while the laccase concentration in the 30% wine lees was enhanced by the addition of 1 mM copper. Although no single factor could be altered in order to enhance enzyme synthesis over a broad spectrum of wine-related wastewaters, multiple doses of 2,5-xylidine could significantly increase laccase synthesis in distillery wastewaters.

## Figures and Tables

**Table 1:** Laccase synthesis with different carbon, nitrogen, lignin/cellulose sources, and phosphorus (*n* = 2).

		HLA* ± std dev (units/L)	Day of HLA	Increase (fold)
Carbon sources	Fructose	3160 ± 179	9 ± 0.6	1.7 ± 0.09
	Glucose	3238 ± 793	9 ± 0.6	1.7 ± 0.42
	Mannitol	1091 ± 161	6 ± 0.0	0.6 ± 0.08
	Maltose	2755 ± 188	9 ± 0.0	1.5 ± 0.10
	Sucrose	3239 ± 781	9 ± 0.6	1.7 ± 0.41
	Cellobiose	3306 ± 262	9 ± 0.0	1.7 ± 0.14
	Lactose	2933 ± 164	10 ± 0.6	1.5 ± 0.09

Nitrogen sources	NH_4_NO_3_	1759 ± 218	6 ± 0.0	0.9 ± 0.11
	NH_4_Cl	1521 ± 290	5 ± 0.0	0.8 ± 0.15
	KNO_3_	1879 ± 313	10 ± 0.0	1.0 ± 0.16
	H_2_NCNH_2_	1485 ± 166	5 ± 0.0	0.8 ± 0.09
	Malt extract	2080 ± 489	10 ± 0.0	1.1 ± 0.26
	Yeast extract	2243 ± 35	5 ± 0.0	1.2 ± 0.02
	Peptone	3428 ± 422	5 ± 0.0	1.8 ± 0.22

Lignin/cellulose	Cellulose	1289 ± 220	8 ± 0.6	0.7 ± 0.12
	Bluegum	1919 ± 206	10 ± 0.0	1.0 ± 0.11
	Rooibos	2031 ± 452	9 ± 0.6	1.1 ± 0.24
	Bagasse	1788 ± 288	9 ± 0.0	0.9 ± 0.15

Phosphorous source	H_3_PO_4_	1978 ± 44	9 ± 1.2	1.0 ± 0.02

	Control	1899 ± 38	10 ± 0.6	1.0 ± 0.02

*HLA: highest laccase activity.

**Table 2:** Laccase synthesis obtained with the addition of various reported inducers prior to inoculation or four days thereafter (*n* = 3).

Reported inducer	Added prior to inoculation			Added after four days		
	HLA* (units/L)	Day of HLA	Increase (fold)	HLA (units/L)	Day of HLA	Increase (fold)
2,5-Xylidine	8419	2	3.7	2944	11	2.4
Ethanol	6701	20	2.9	292	6	0.2
Copper	5492	20	2.4	1044	13	0.9
4-Methylcatechol	1153	20	0.5	2253	13	1.9
*n-hydroxyphthalimide*	1636	14	0.7	2283	13	1.9
Gallic acid	3303	14	1.4	820	8	0.7
Tannic acid	3114	20	1.4	1111	13	0.9
Quercetin	2966	14	1.3	588	13	0.5
Syringic acid	1862	14	0.8	1404	11	1.2
Guaiacol	2580	16	1.1	1292	11	1.1
Dimethoxybenzyl alcohol	1939	16	0.8	1247	13	1.0
Phenol	2149	14	0.9	1270	11	1.0
Violuric acid	2035	14	0.9	1039	11	0.9
Phenol red	2601	12	1.1	663	13	0.5
Cellulose	2474	16	1.1	328	13	0.3
*p*-Coumaric acid	2370	14	1.0	1227	13	1.0
Rooibos	2119	16	0.9	372	13	0.3
*o*-Cresol	2064	12	0.9	558	11	0.5
Dichloroindophenol	127	12	0.1	795	8	0.7
Hydroxybenzotriazole	977	14	0.4	656	13	0.5
Cycloheximide	20	4	0.0	455	5	0.4
Control	2305	12	1.0	1214	13	1.0

*HLA: highest laccase activity.

**Table 3:** Laccase synthesis when varying 2,5-xylidine dosage time and number (*n* = 2).

Days added	HLA (units/L)	Std Dev	Day of HLA	Std Dev	Increase (fold)
Control	1295	152	13	1	1.0

0	9999	411	2	0	7.7
0 + 2	11012	688	3	1	8.5
0 + 2 + 4	13294	627	7	1	10.3

2	7529	321	4	0	5.8
2 + 4	7589	346	4	1	5.9
2 + 4 + 6	7817	661	4	1	6.0

4	1972	323	6	0	1.5
4 + 6	3579	417	9	1	2.8
4 + 6 + 8	5033	327	13	2	3.9

6	4058	366	11	1	3.1
6 + 8	3399	192	9	1	2.6
6 + 8 + 10	3930	298	9	1	3.0

8	1454	154	13	1	1.1
10	1134	78	13	0	0.9

*HLA: highest laccase activity.

**Table 4:** Wastewater characteristics [9].

	pH	COD (g/L)	Total phenols (mg/L)
Brandy distillery wastewater	3.67	19.9	320
Distilled wine	3.58	34.8	290
Distilled wine lees	5.09	45.5	540
Wine lees	3.72	211.8	1720

## References

[1] Gianfreda L, Xu F, Bollag JM (1999). Laccases: a useful group of oxidoreductive enzymes. *Bioremediation Journal*.

[2] Strong PJ, Claus H (2011). Laccase: a review of its past and its future in bioremediation. *Critical Reviews in Environmental Science and Technology*.

[3] Couto SR, Gundín M, Lorenzo M, Sanromán MÁ (2002). Screening of supports and inducers for laccase production by Trametes versicolor in semi-solid-state conditions. *Process Biochemistry*.

[4] Gómez J, Pazos M, Couto SR, Sanromán MA (2005). Chestnut shell and barley bran as potential substrates for laccase production by Coriolopsis rigida under solid-state conditions. *Journal of Food Engineering*.

[5] Osma JF, Toca Herrera JL, Rodríguez Couto S (2007). Banana skin: a novel waste for laccase production by Trametes pubescens under solid-state conditions. Application to synthetic dye decolouration. *Dyes and Pigments*.

[6] Osma JF, Saravia V, Herrera JLT, Couto SR (2007). Mandarin peelings: the best carbon source to produce laccase by static cultures of Trametes pubescens. *Chemosphere*.

[7] Rosales E, Couto SR, Sanromán MA (2005). Reutilisation of food processing wastes for production of relevant metabolites: application to laccase production by Trametes hirsuta. *Journal of Food Engineering*.

[8] Rodríguez Couto S, López E, Sanromán MA (2006). Utilisation of grape seeds for laccase production in solid-state fermentors. *Journal of Food Engineering*.

[9] Strong PJ, Burgess JE (2008). Fungal and enzymatic remediation of a wine lees and five wine-related distillery wastewaters. *Bioresource Technology*.

[10] Shuttleworth KL, Bollag JM (1986). Soluble and immobilized laccase as catalysts for the transformation of substituted phenols. *Enzyme and Microbial Technology*.

[11] Alves AMCR, Record E, Lomascolo A (2004). Highly efficient production of laccase by the basidiomycete Pycnoporus cinnabarinus. *Applied and Environmental Microbiology*.

[12] Galhaup C, Goller S, Peterbauer CK, Strauss J, Haltrich D (2002). Characterization of the major laccase isoenzyme from Trametes pubescens and regulation of its synthesis by metal ions. *Microbiology*.

[13] Galhaup C, Wagner H, Hinterstoisser B, Haltrich D (2002). Increased production of laccase by the wood-degrading basidiomycete Trametes pubescens. *Enzyme and Microbial Technology*.

[14] Revankar MS, Lele SS (2006). Increased production of extracellular laccase by the white rot fungus Coriolus versicolor MTCC 138. *World Journal of Microbiology and Biotechnology*.

[15] Fåhraeus G, Reinhammar B (1967). Large scale production and purification of laccase from cultures of the fungus Polyporus versicolor and some properties of laccase A. *Acta chemica Scandinavica*.

[16] Pointing SB, Jones EBG, Vrijmoed LLP (2000). Optimization of laccase production by Pycnoporus sanguineus in submerged liquid culture. *Mycologia*.

[17] Collins PJ, Dobson ADW (1997). Regulation of laccase gene transcription in Trametes versicolor. *Applied and Environmental Microbiology*.

[18] Van Heerden FR, Van Wyk BE, Viljoen AM, Steenkamp PA (2003). Phenolic variation in wild populations of Aspalathus linearis (rooibos tea). *Biochemical Systematics and Ecology*.

[19] Rabe C, Steenkamp JA, Joubert E, Burger JFW, Ferreira D (1994). Phenolic metabolites from rooibos tea (Aspalathus linearis). *Phytochemistry*.

[20] Xu F, Sun RC, Sun JX, Liu CF, He BH, Fan JS (2005). Determination of cell wall ferulic and p-coumaric acids in sugarcane bagasse. *Analytica Chimica Acta*.

[21] D’Souza DT, Tiwari R, Sah AK, Raghukumar C (2006). Enhanced production of laccase by a marine fungus during treatment of colored effluents and synthetic dyes. *Enzyme and Microbial Technology*.

[22] Crestini C, D’Annibale A, Giovannozzi-Sermanni G (1996). Aqueous plant extracts as stimulators of laccase production in liquid cultures of Lentinus edodes. *Biotechnology Techniques*.

[23] Fåhraeus G, Tullander V, Ljunggren H (1958). Production of high laccase yields in cultures of fungi. *Physiologia Plantarum*.

[24] Lomascolo A, Record E, Herpoël-Gimbert I (2003). Overproduction of laccase by a monokaryotic strain of Pycnoporus cinnabarinus using ethanol as inducer. *Journal of Applied Microbiology*.

[25] Meza JC, Auria R, Lomascolo A, Sigoillot JC, Casalot L (2007). Role of ethanol on growth, laccase production and protease activity in Pycnoporus cinnabarinus ss3. *Enzyme and Microbial Technology*.

[26] Bollag JM, Leonowicz A (1984). Comparative studies of extracellular fungal laccases. *Applied and Environmental Microbiology*.

[27] Tavares APM, Coelho MAZ, Coutinho JAP, Xavier AMRB (2005). Laccase improvement in submerged cultivation: induced production and kinetic modelling. *Journal of Chemical Technology and Biotechnology*.

[28] Eggert C, Temp U, Dean JFD, Eriksson KEL (1996). A fungal metabolite mediates degradation of non-phenolic lignin structures and synthetic lignin by laccase. *FEBS Letters*.

[29] Strong PJ, Burgess JE, Chamy R, Ruiz G, Carballa M (2007). Bioremediation of a wine distillery wastewater using white rot fungi and the subsequent production of laccase. *Water Science and Technology*.

[30] Moreira MT, Palma C, Feijoo G, Lema JM (1998). Strategies for the continuous production of ligninolytic enzymes in fixed and fluidised bed bioreactors. *Journal of Biotechnology*.

[31] Neifar M, Kamoun A, Jaouani A (2011). Application of Asymetrical and Hoke designs for optimization of laccase production by the white-rot fungus Fomes fomentarius in solid-state fermentation. *Enzyme Research*.

